# Biochar compost blends facilitate switchgrass growth in mine soils by reducing Cd and Zn bioavailability

**DOI:** 10.1007/s42773-019-00004-7

**Published:** 2019-03-29

**Authors:** Jeffrey M. Novak, James A. Ippolito, Donald W. Watts, Gilbert C. Sigua, Thomas F. Ducey, Mark G. Johnson

**Affiliations:** 1 Coastal Plains Soil, Water and Plant Research Center, United States Department of Agriculture, Agricultural Research Service, Florence, SC 29501, USA; 2 Department of Soil and Crop Sciences, Colorado State University, Fort Collins, CO 80523, USA; 3 National Health and Environmental Effects Research Laboratory, United States Environmental Protection Agency, Corvallis, OR 97331, USA

**Keywords:** Biochar, Heavy metals, Metal sorption, Tri-State Mine soil, Remediation, Switchgrass

## Abstract

Biochars have the potential to reclaim mine-impacted soils; however, their variable physico-chemical properties incite speculation about their successful remediation performance. This investigation examined the capability of biochars produced from three different feedstocks along with a compost blend to improve switchgrass growth conditions in a mine-impacted soil by examining influences on soil pH, grass metal contents, and soil-extractable metal concentrations. Cadmium (Cd)- and zinc (Zn)-contaminated mine soil was collected from a site near Webb City, Missouri, USA—a location within the Tri-State Mining District. In a full factorial design, soil was treated with a 0%, 2.5%, and 5% (*w/w*) compost mixture (wood chips + beef cattle manure), and 0%, 2.5% and 5% of each biochar pyrolyzed from beef cattle manure, poultry litter, and lodgepole pine feedstocks. Switchgrass (*Panicum virgatum*, ‘Cave-In-Rock’ variety) was grown in a greenhouse for 50 days and the mass of shoots (above-ground biomass) and roots was assessed, while soil pH, deionized H_2_O- and 0.01 M CaCl_2_-extractable Cd and Zn concentrations were measured. Poultry litter biochar and compost had the greatest ability to raise soil pH (from 4.40 to 6.61), beef cattle manure biochar and compost moderately raised pH (from 4.4 to 5.92), and lodgepole pine biochar and compost weakly raised pH (from 4.40 to 5.05). Soils treated with beef cattle manure biochar, poultry litter biochar significantly reduced deionized H_2_O- and 0.01 M CaCl_2_-extractable Cd and Zn concentrations, while lodgepole pine biochar-treated soils showed mixed results. Switchgrass shoot and root masses were greatest in soil treated with compost in combination with either beef cattle manure biochar or poultry litter biochar. Soils treated with 5% beef cattle manure biochar + 5% compost had greater reductions in total Cd and Zn concentrations measured in switchgrass shoots and roots compared to the other two treatments. The three biochars and compost mixtures applied to heavy metal, mine-impacted soil had considerable performance dissimilarities for improving switchgrass productivity. Switchgrass growth was noticeably improved after treatment with the compost in combination with biochar from beef cattle manure or poultry litter. This may be explained by the increased soil pH that promoted Zn and Cd precipitation and organic functional groups that reduced soil-available heavy metal concentrations. Our results imply that creating designer biochars is an important management component in developing successful mine-site phytostabilization programs.

## 1 Introduction

Mining activities and ensuing disposal of waste products can have profound impacts on soil health characteristics (low pH, toxic heavy metals concentrations, etc.) where mine wastes are stored. Reports have revealed that milling operations, rock grinding, concentrating ores processes, and disposal of sulfurous tailings can lower soil pH (Dudka and Adriano 1997; Novak et al. 2018), create poor microbial habitat conditions (Cui et al. 2013; Hu et al. 2014), reduce soil microbial enzyme activity (Hanauer et al. 2012; Nie et al. 2018; Novak et al. 2018), and contribute to toxic metal concentrations in soils (Kabata-Pendias 2001; Schreurs et al. 2011). Stabilizing mine tailings and mine-impacted soils with a ground cover is an important management practice because plants can minimize off-site movement of toxic metals and can add organic matter to improve soil chemical characteristics for better plant growth (Brown et al. 2003b; Figueroa et al. 2010; Maiti and Maiti 2014). Without treatment, however, these poor soil: plant growth characteristics are known to impair ground cover establishment and hence influence the degree of site phytostabilization (Phillips et al. 2016; Rizwan et al. 2016; Kumar et al. 2018). Thus, in mine soil remediation, a key goal is to reduce heavy-metal bioavailability while also improving soil health characteristics (i.e., pH, nutrient availability, etc.) that are conducive for plant growth. To achieve this remediation goal, various materials are often utilized as amendments.

An assortment of conventional materials has been previously employed on mine spoils or mine-impacted soils to modify soil chemical issues and enhance phytostabilization. They include, additions of C-based materials to bolster soil organic matter levels (Norland 1993); adding lime to raise pH (Hensley and Carpenter 1984; Srivastava and Chhonkur 2000); adding municipal biosolids (Haering et al. 2000; Brown et al. 2003a; Waqas et al. 2014); or introducing wood pulp sludge and/or fly ash (Li and Daniels 1997; Gorman et al. 2000; Abbot et al. 2001) to bind metals. Most of these amendments are effective at ameliorating soil: plant chemical issues including raising pH and sorbing toxic metals. Novak et al. (2018) raised concern, however, that these conventional amendments were not long lasting, required high application rates, and/or could produce malodor issues.

In place of these amendments and their noted shortcomings, current literature adjudicates the use of biochar as an amendment in mine reclamation sites (Ippolito et al. 2017; Kumar et al. 2018; O’Connor et al. 2018). Biochar is now the in-vogue amendment for mine-site restoration because previous research has shown that biochars can bind with heavy metals (Ehsan et al. 2014; Ippolito et al. 2017) as well as improve soil health characteristics by acting as a liming agent (Yuan and Xu 2011; Jian et al. 2014), rebuilding organic matter levels (Brockhoff et al. 2010; Anawar et al. 2015), supplying plant nutrients (Novak et al. 2009; Ippolito et al. 2015; Ahmad et al. 2018) and stimulating soil microbial activity (Ducey et al. 2013). The ability of biochars to reduce bioavailable forms of toxic soil metals is attributed to various immobilization mechanisms, including adsorption, precipitation, and complexation reactions (Rizwan et al. 2016; Ippolito et al. 2017; Wang et al. 2018).

Biochar is a material produced by pyrolysis of organic feedstocks under anoxic conditions using temperatures ranging from 350 to 900 °C (Laird et al. 2009; Boateng et al. 2015). A variety of feedstocks is available for biochar production including agricultural by-products (i.e., nut shells, peach pits, etc.), forestry residues, wood wastes, crop residues, animal manures, and municipal biosolids (Cantrell et al. 2012; Spokas et al. 2012; Zheng et al. 2013). Reports have shown that the variety of feedstocks, pyrolysis conditions, and biochar supply chain management options contributes to diversity in their chemical and physical properties (Novak et al. 2013; Liu et al. 2015; Anderson et al. 2016). Other investigations corroborate that biochars can have differences in their pH, ash contents, C/N, O/C, and H/C ratios, porosity, specific surface area, cation exchange capacity, surface charge, OH- and COOH-containing functional groups, and aromatic C–C double bonds that will influence their propensity to react with toxic metals and improve soil chemical conditions (Guo et al. 2015; Sizmur et al. 2015; Xie et al. 2015).

The inherent variability in biochars chemical and structural properties along with diverse characteristics of metal-contaminant characteristics (i.e., valency, *K*_sp_, etc.), calls attention to potential performance differences in mine-site remediation ventures (Fellet et al 2011; Kelly et al. 2014). Moreover, poor-performing biochar applied to mine spoils for remediation purposes cannot be physically removed nor economically substituted with another biochar. Thus, it is prudent to perform detailed studies to pre-select a biochar that has a high level of certainty of achieving the desired site remediation results while not negatively impacting soil health characteristics nor facilitating off-site movement of toxic metals.

Therefore, it was our intention to improve the knowledge pool of how biochars produced from diverse feedstocks (poultry litter, lodgepole pine, beef cattle manure) would influence switchgrass (*Panicum virgatum*) shoot and root growth, metal uptake, while also improving common soil health characteristics (pH, reducing toxic heavy metal bioavailability) in a mine-impacted soil. Along with these biochars, a compost mixture (beef cattle manure + wood chips) was also supplemented with the biochars to potentially stimulate soil microbial activity and enzyme production, which are indicators of soil health status (Cardoso et al. 2013). Mine-impacted soil was obtained from a EPA superfund site known to contain high concentrations of Cd and Zn (Johnson et al. 2016). Our hypothesis was that these three biochars would have distinct, but contrary, performance abilities at site remediation to facilitate switchgrass growth and bioavailability of Cd and Zn concentrations. Ultimately, these data will be utilized for actual on-site remediation.

## 2 Material and methods

### 2.1 Site, soil description, preparation, and characterization

A metal-contaminated field was selected near Webb City in Jasper County, Missouri, USA (latitude 37.129272, longitude – 94.447939). This location is part of the Oronogo-Duenweg mining area of the Tri-State Mining District located in Southwest Missouri. Mining of lead (Pb) and zinc (Zn) ore has occurred across this portion of the District with the mining field encompassing approximately 49,800 ha (Johnson et al. 2016). The mine waste ores and tailings were stored in chat piles near production centers (Gibson 1972). The chat piles contain residual Pb, Cd, and Zn concentrations, which in some locations leached into the underlying soil or were carried away as dust by winds (EPA 2018).

Potential exposure of Pb- and Zn-contaminated chat raises human health concerns, so it was prudent to excavate and remove the chat (Johnson et al. 2016). During chat and contaminated soil removal, subsoil was exposed at the surface (Fig. S1). The subsoil was composed of a heterogeneous texture consisting of extremely gravelly silt loam, cobbly clays, which were integrated with cherty-to-limestone rock fragments ranging in size from 2 to 15-cm in diameter (Fig. S1; Soil Survey of Jasper County, Missouri, 2002). The exposed subsoil was leveled across the field with the intent of re-seeding with native grasses. For our purposes, a backhoe was used to collect a few hundred kg of B/C sub horizon material (See Fig. S1). The subsoil was placed in plastic-lined metal drums and transported to the US Department of Agriculture-Agricultural Research Service-Florence, South Carolina, USA location (USDA-ARS-Florence).

Once in Florence, the subsoil material was air-dried, and screened using a 12.7-cm diameter sieve to collect soil material more appropriate for use in a greenhouse pot experiment. Sieving the soil revealed that it contained approximately 30% (w/w) coarse fragments that were > 12.7-cm in diameter. Air-dried soil that passed through the sieve was stored in plastic-lined drums for characterization and for future use in the greenhouse experiment.

The sieved soil was characterized for pH using a 1:2 (w/w) soil-to-deionized water ratio. Additionally, metal concentrations were extracted using deionized water, bioavailable metals were extracted using 0.01 M CaCl_2_ (Ippolito et al. 2017), and total metal concentrations were estimated after digestion with 4 M HNO_3_ (Bradford et al. 1975). Both deionized water and 0.01 M CaCl_2_ metal concentrations were determined in triplicate by extracting 30 g soil with 60 mL of extractant, shaken for 30 min, and filtered using a nylon 0.45 μM filter syringe. Total metal concentrations were determined in triplicate by digestion of 10 g soil in 100 mL of 4 M HNO_3_. All metal concentrations were quantified via inductively coupled plasma-optical emission spectroscopy (ICP-OES) and the data are presented in Table S1.

### 2.2 Amendment collection, preparation, pyrolysis, and characterization

Three feedstocks were used to produce biochars in this experiment. Raw beef cattle manure from a local feedlot operation near Webb City, Missouri, USA was collected from a stockpile (latitude 37.14522, longitude – 94.45206). The manure was mixed with local wood chips (50:50 mixture, w/w) and the blend was exposed to the environment for 2–3 years to allow for conversion into a more degraded manure/compost mixture (George King, personal communication, 2015). A few kg of the manure compost was transported to the USDA-ARS-Florence location and passed through a 6-mm sieve. The 6-mm sieved beef cattle manure compost was split into two portions—one portion was pyrolyzed at 500 °C into biochar as outlined (Novak et al. 2014) and the remaining portion was stored for later use in the greenhouse experiment (Sect. 2.3). The remaining two biochars were available commercially and consisted of biochar produced from poultry litter and lodgepole pine feedstocks. The lodgepole pine biochar was produced using a two-stage process as described by Ippolito et al. (2017). Briefly, in the first stage, the feedstock was initially held between 500 and 700 °C for < 1 min under a very low O_2_ atmosphere. In the second stage, the material was pyrolyzed between 300 and 550 °C for approximately 15 min hold time in an anaerobic environment. After pyrolysis, the lodgepole pine biochar was cooled, and then passed through a 0.25-mm sieve. The poultry litter biochar was produced by gasification using a fixed-bed pyrolyzed programed for conditions (temperature and hold time) that are propriety.

All three biochars and the compost were characterized for their pH in a 1:2 (w/w) biochar or compost-to-deionized water ratio (Novak et al. 2014). All three biochars were also characterized by ultimate analysis (ASTM D 3176; Hazen Research, Inc., Golden, Colorado, USA) for their ash, C, H, O, N and S contents. Their molar H/C and O/C ratios were calculated from the elemental analysis (Table S2). Total elemental composition of the ash fraction in the three biochar feedstocks and the manure compost was determined by first ashing the samples at 600 °C, digesting the ash using method SW866 (ASTM 2006), and then quantifying metal content using ICP-OES by Hazen Research, Inc. The chlorine and silica contents in the ash fraction of these four materials were determined using methods ASTM D 2361 and ASTM D 5865, respectively (ASTM 2006). Elemental compositional analysis in the ash fraction is reported in Table S2.

### 2.3 Greenhouse experiment

In a factorial design, the treatments for the switchgrass greenhouse experiment consisted of mine soil mixed with biochar at 0%, 2.5%, and 5%, and then blended with compost at 0%, 2.5%, and 5% (w/w; Table S3). Compost produced from beef cattle manure and wood chips was included as a C source to stimulate microbial activity. Materials were added to 1500 g of air-dried, 12.7-mm sieved mine soil. After fully hand incorporating the amendments, sufficient deionized water was added to bring the soil gravimetric moisture content to 15% (w/w) on an air-dry basis. Preliminary experiments showed that at greater water contents (> 15%), homogeneous dispersing of the amendments into the mine soil was difficult due to clumping.

The treated and untreated mine soils were placed into triplicate plastic pots (15-cm top diameter × 17-cm deep) and gently tapped to a bulk density of 1.5 g/cm^3^ as outlined by Novak et al. (2018). About 40 switchgrass seeds were then planted in each pot to a 1-cm depth. The pots were transported to a greenhouse and randomly placed on benches.

The pots remained in the greenhouse for 50 days under a mean air temperature of 29.1 °C (SD 3.3) and mean relative humidity of 81% (SD 9.4). On day 16, all pots were fertilized with a 30 mL solution of NH_4_NO_3_ that delivered an equivalent of 3 kg N/ha because some treatments exhibited a N-deficient (i.e., chlorosis) symptom. No inorganic P or K was added to the pots because these nutrients were supplied with the amendments (Table S2). The pots were irrigated by hand using tap water several times per week over the 50-d experiment.

At termination (day 50), stems and roots were harvested from each pot and oven-dried (60 °C). Plant samples were then digested in a hot block acid digestor using concentrated HNO_3_ at 60 °C for 30 min followed by more digestion at 90 °C for 90 min with the addition of 30% H_2_O_2_ as described by Huang and Schulte (1985). The total Cd and Zn concentrations in the digested switchgrass shoots and roots were quantified using ICP-OES. The remaining soils in the pots were oven-dried (105 °C) and then subsamples extracted using deionized H_2_O, 0.01 M CaCl_2_, and extractable Cd and Zn in each extract was quantified using ICP-OES.

### 2.4 Statistics

Biochar and compost were the fixed factors, while the mean switchgrass shoot and root masses, total Cd and Zn concentrations in roots and stems along with soil pH, deionized water, 0.01 M CaCl_2_ extractable Cd and Zn concentrations were the dependent variables in the statistical analysis employing a two-way ANOVA with significance determined at a *P* < 0.05. All statistics were completed using Sigma Stat v. 3.5 software (SSPS Corp., Chicago, IL, USA).

## 3 Results and discussion

### 3.1 Mine soil and biochar characteristics

Copper and Zn are common constituents in the soils’ inorganic mineral composition. The Cu and Zn concentration in loamy-to-clay-textured soils of the USA can range from 7 to 70 and 20 to 220 mg/kg, respectively (Kabata-Pendias 2001). With respect to Cd, its contents in loamy and clay-textured soils in the USA can range from 0.13 to 0.55 mg/ kg (Kabata-Pendias 2001). In heavily Cd-contaminated soils, Riley et al. (1992) reported that concentrations can be as high as 345 mg/kg. Cd in soils is regarded as a toxic compound to plants and humans (Wauana and Okieimen 2011). Here, soil material collected at the mine site had a pH of 4.4 and contained elevated concentrations of total Cu, Cd, and Zn (66.5, 72.2 and 2225 mg/kg, respectively; Table S1). On a positive soil fertility note, this soil contains sufficient total concentrations of important plant nutrients such as K, Mg, and P.

Examining the total, water, and bioavailable concentrations of Cu, Cd, and Zn revealed interesting variance between metal availability and binding sites. For example, water and bioavailable Cu concentrations were < 2.2 mg/kg, implying most of soil Cu is bound to phases that are not easily removable/bioavailable. In contrast, there were much larger water and bioavailable Zn and Cd concentrations compared to Cu. These Cd and Zn fractions were probably bound to easily soluble salts and as exchangeable phases on solids (e.g., clays) and organic materials (Beesley and Marmiroli 2011). On a positive note, there was < 0.5 mg/kg of other water soluble and bioavailable metals such as Cr, Ni, and Pb concentrations in this soil (Table S1).

Biochar and compost made from beef cattle manure had relatively medium–high pH values (6.8 and 9.5, respectively), but were strikingly low in C content (Table S2). The C content was low in this feedstock due to exposure to weathering conditions and microbial mineralization during the long-term (2–3 years) composting process. With the loss of C, it should be expected that the ash contents of a manure-based compost would be relatively high (68.4%–83.1%, Table S2; Cantrell et al. 2012). Moreover, their ash is dominated by Si (> 77%), with lower contents of Al (3%, Table S2). These inorganic chemical characteristics imply that soil was included during beef cattle manure feedstock collection from the cattle yard. Others have shown that it is not unusual for biochars produced from animal manure feedstocks to contain high ash contents (50% to > 70%, Enders et al. 2012; Gunamantha and Widana, 2018).

Biochar produced from lodgepole pine and poultry litter feedstocks both had alkaline pH values (pH > 9, Table S2) which is typical for these two feedstocks (Novak et al. 2013; Ippolito et al. 2015). The C content was also much higher for lodgepole pine biochar compared to poultry litter biochar, but poultry litter biochar is enriched with plant nutrients such as N, P, and K. Striking results were also noted for the ash compositional differences between these two feedstocks (3.2% vs. 42.5%, Table S2). The plant nutrient compositional differences are consistent with other compositional results in biochar produced from lodgepole pine (Robertson et al. 2012) and poultry litter feedstock (Cantrell et al. 2012; Wang et al. 2015).

### 3.2 Soil characteristics after biochar treatment

**Fig. 1:**
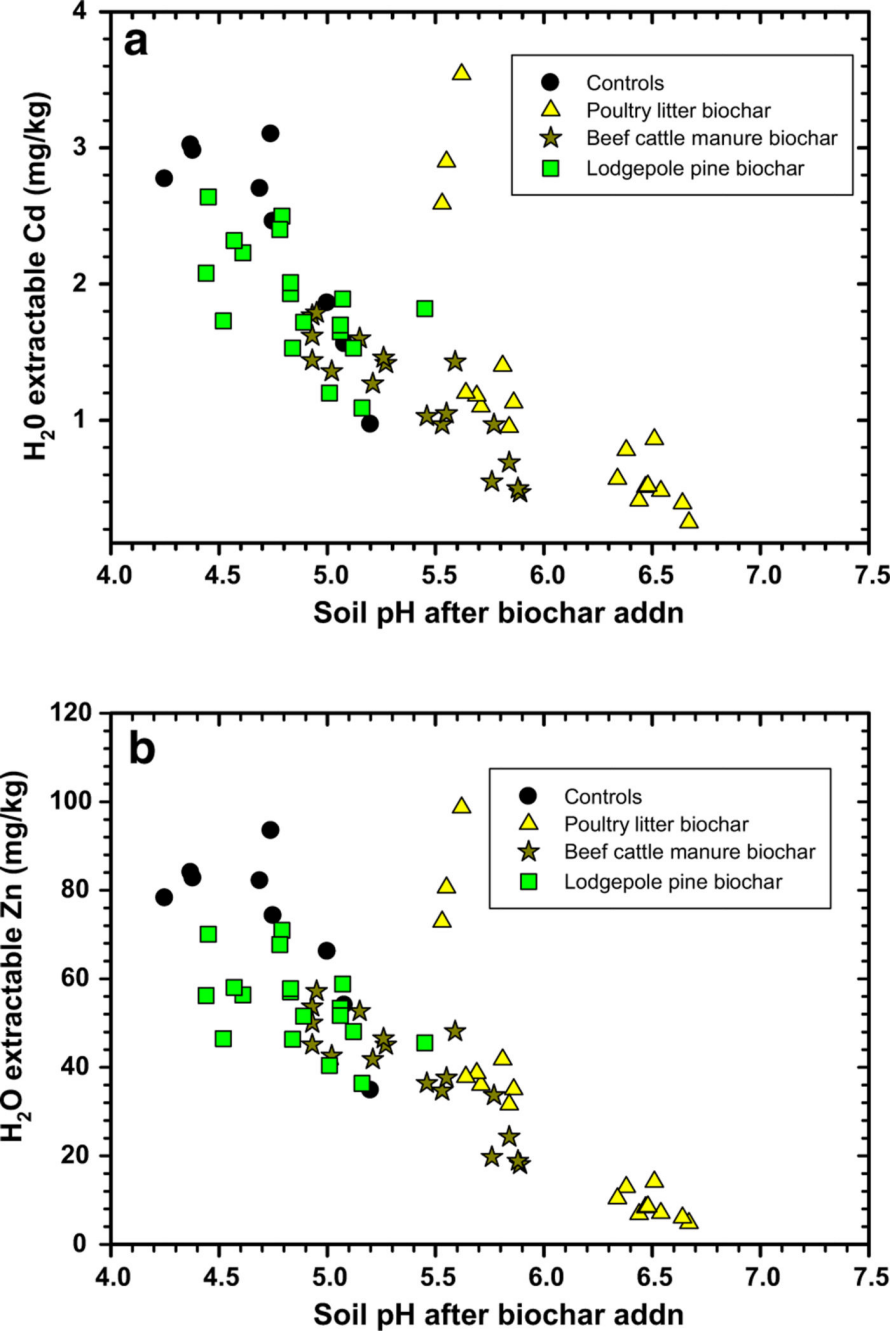
Water-extractable cadmium (Cd, **a**) and zinc (Zn, **b**) from compost + biochar-treated Tri-State Mine soil versus their pH measured at end of study

Soil pH increases are important to achieve in acidic mine soil remediation because metal solubility is reduced under alkaline conditions (Bohn et al. 1979; Kabata-Pendias, 2001). Our results corroborate this fact, after measuring the extractability of Cd and Zn concentrations in all (Fig. 1a, b). Addition of poultry litter and compost increased soil pH, which concomitantly reduced H_2_O extractable Cd and Zn concentrations (Table 1). Moderate reductions in H_2_O-extractable Cd and Zn were obtained using beef cattle manure and compost (Fig. 1a, b and Table 1) followed by minimal reductions after addition of lodgepole pine biochar and in controls without compost. We suspect that the lodgepole pine biochar did not impact soil pH to the same degree as the other two biochars as noted in Fig. 1a, b. In soils treated with lodgepole pine biochar and ± compost, the pH values were < 5.5 resulting in more soluble forms of Cd and Zn available for extraction. It can also be speculated that the poultry litter biochar was more effective at binding H_2_O soluble Cd and Zn because of the presence of ligand sites on the numerous C-assemblies of structures (Guo et al. 2009) and sorption by its mineral ash constituents (Ahmad et al. 2014).

In Fig. 1a, b, three H_2_O-extractable Cd and Zn outliers occurred in soils treated with 2.5% poultry litter biochar and 0% compost, suggesting that compost is necessary to further reduce these two metal concentrations. Without biochar or compost additions, the soil pH values remain acidic (pH < 5) resulting in higher H_2_O-extractable Cd (2.5–3.1 mg/kg) and Zn (75–95 mg/kg, Fig. 1). Among the three biochar amendments, application of poultry litter and compost resulted in the largest increase in mine soil pH (from 4.40 to 6.61; Table 2). Beef cattle manure biochar plus compost showed a moderate soil pH change from 4.40 to 5.92, while lodgepole pine biochar additions produced the lowest change from 4.40 to 4.81. Statistical analysis revealed that significant pH modifications occurred after biochar and compost addition and their interactions were significant (Table 2). However, an additional finding was revealed when choosing organic amendments in mine soils/spoils remediation—the amendments should have a significant liming capacity. Thus, organic amendments should be able to raise spoil/soil pH levels to be > 5 to reduce water-soluble Cd and Zn concentrations.

**Fig. 2:**
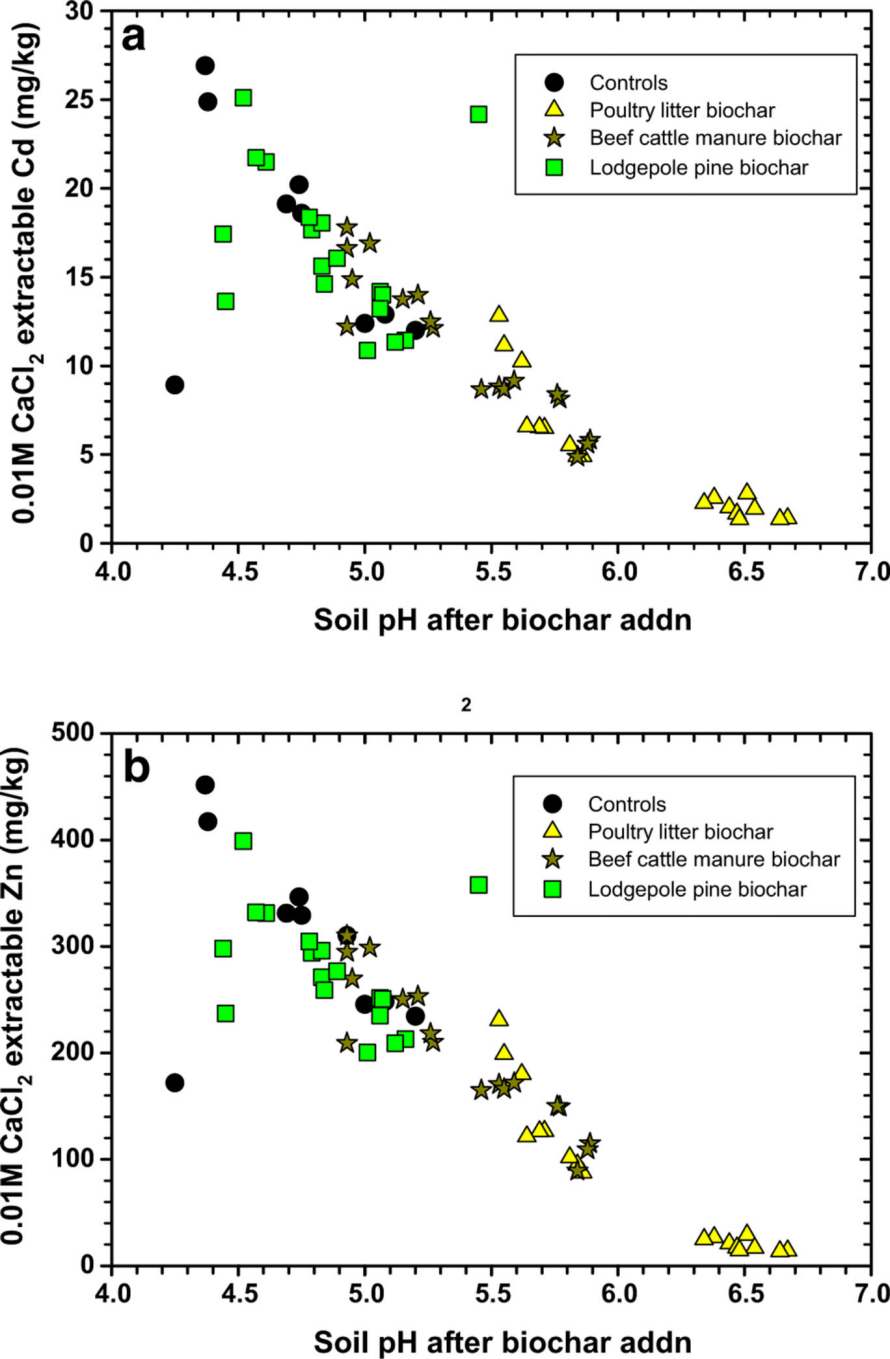
0.01 M CaCl_2_-extractable cadmium (Cd, **a**) and zinc (Zn, **b**) from compost + biochar-treated Tri-State Mine soil versus their pH measured at end of study

Metals in soils are also extracted using dilute salt solutions such as 0.01 M CaCl_2_ (salt) that by convention represents the bioavailable fraction (Kim et al. 2015; Ippolito et al. 2017). The bioavailable fraction implies that these metals are plant-available and that they are easily exchangeable with metals held electrostatically on charged surfaces, other salts, and organic ligands. The largest reduction in bioavailable Cd and Zn occurred with addition of 5% poultry litter biochar and compost, with mean concentrations being significantly reduced from 20.2 to 1.4 mg/kg for Cd, and 346 to 14 mg/kg for Zn compared to the control (or 93% and 96%, reductions, respectively; Table 3). Treatment with beef cattle manure biochar was also capable of reducing both bioavailable Cd and Zn concentrations, and a greater reduction occurred when this biochar was mixed into the soil with compost (Table 3). On the opposite side, treatment with lodge pole pine biochar was not as effective compared to the other two biochars at reducing bioavailable Cd and Zn concentrations (Table 3). The reductions in bioavailable Cd and Zn based on lodgepole pine biochar alone were not significant as compared to the control (*P* < 0.27; Table 3); however, adding compost did improve the level of reduction (*P* < 0.01, Table 3). In all treatments, the interaction between biochar and compost was not significant for bioavailable Cd and Zn (Table 3). The plots of both bioavailable Cd and Zn as a function of soil pH (Fig. 2a, b) revealed trends that were like those found with water extractable metals (Fig. 1). In these cases, raising soil pH after biochar additions produced from poultry litter and beef cattle manure reduced bioavailable Cd and Zn, while lodgepole pine biochar was not as effective at these reductions.

### 3.3 Switchgrass growth in treated and untreated soil

**Fig. 3:**
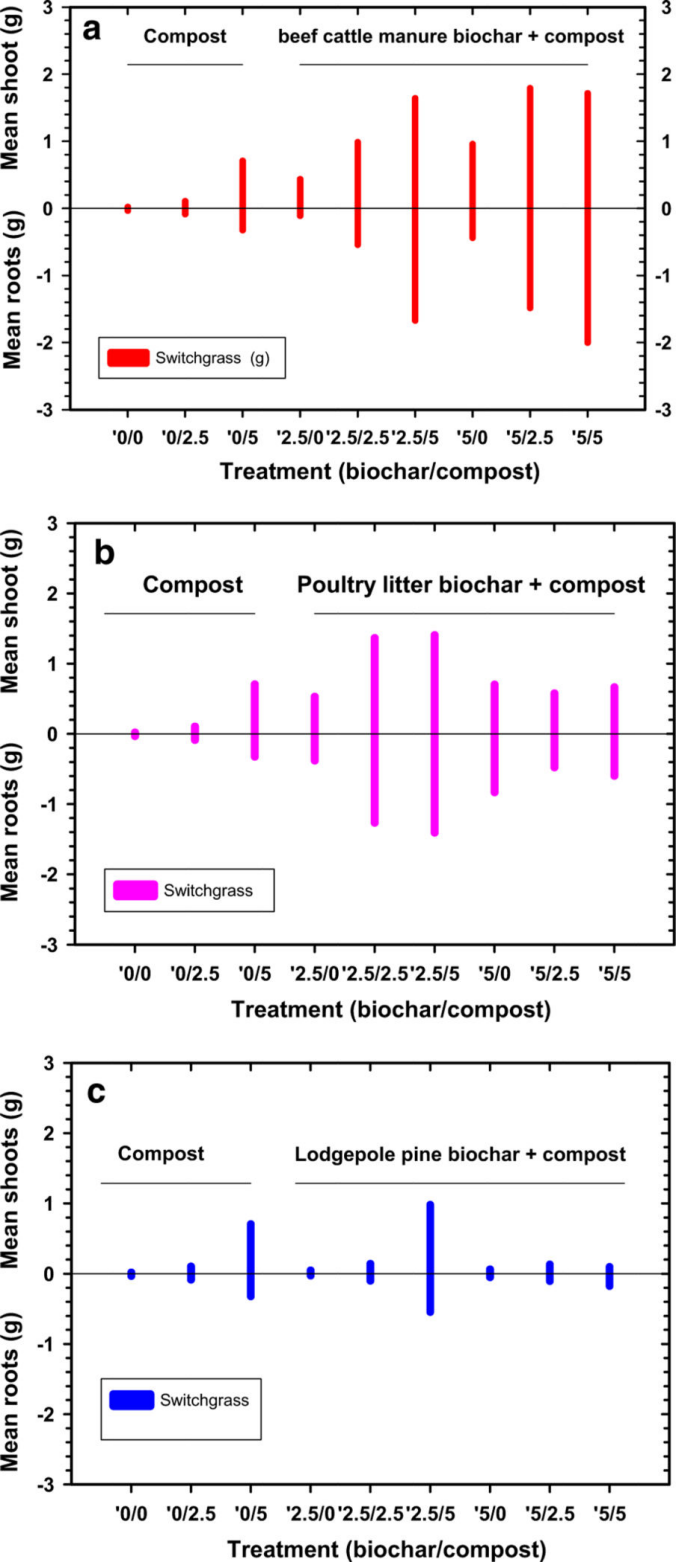
Mean switchgrass shoot/root masses grown in Tri-State Mine soil treated with compost and beef cattle manure biochar (**a**), poultry litter biochar (**b**), and lodgepole pine biochar (**c**) (treatments expressed as biochar%/compost%)

Mean switchgrass above-ground biomass (shoots) and below ground biomass (roots) in the control (0% biochar/0% compost) was poor (Fig. 3a–c). This showed that native soil conditions are inhospitable for switchgrass growth. In contrast, switchgrass growth was improved after treatment with 5% compost (no biochar) that resulted in a small, yet significant increase in mean switchgrass shoot masses (Table 4). The influence on switchgrass root growth in soils treated with compost alone was not as clear. These results indicate that compost alone at 5% application had a significant, but lower impact on improving soil conditions that can positively influence switchgrass shoot and root mean mass results.

Mixed results were observed after mixing in the biochars alone (except lodgepole pine biochar) on mean switchgrass shoot and root masses (Table 4). When compost was added to the biochar treated soils, there were additional significant improvements in switchgrass shoots and root masses (except with poultry litter biochar, Table 4), with similar effects observed by others when utilizing biochar and manures (Lentz and Ippolito 2012). In most cases, the interaction between biochar and compost was significant on switchgrass root and shoot growth. When averaged across biochar and compost treatments, increasing additions of beef cattle manure and poultry litter biochar along with compost addition favored significant increases in switchgrass shoot and root masses with the largest increases occurring at the 5% application rates (Fig. 3; Table 4). Poorer switchgrass shoots and root mean masses occurred in soil treated with lodgepole pine biochar and compost (Fig. 3; Table 4).

**Fig. 4:**
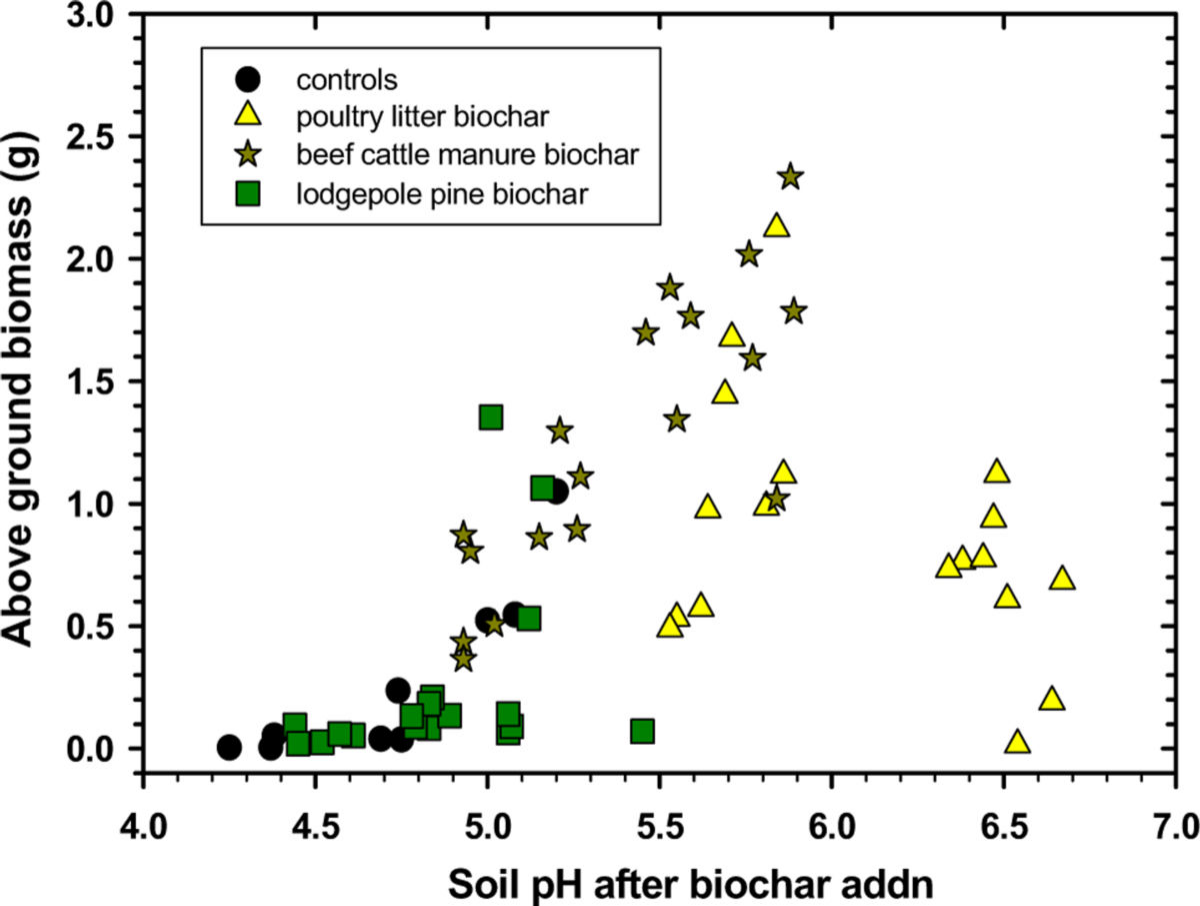
Switchgrass above-ground biomass versus Tri-State Mine soil pH measured after compost and biochar additions

Plotting the switchgrass above-ground biomass (shoots) versus soil pH (Fig. 4) partially corroborated the individual impacts of biochar and compost on mean shoot and root masses as shown in Table 4. The soil controls treated with 0% biochar and 0% or with 2.5% compost alone had minimal switchgrass shoot growth. In comparison, simply adding a larger amount of compost (5%) to the soil controls significantly raised soil pH and resulted in greater shoot mass production. In general, this agrees with statistical results present in Table 4. Similarly, just treating soil with lodgepole pine biochar and 0–2.5% compost showed low soil pH responses that kept switchgrass shoot production at a minimum. Mixing in beef cattle manure biochar along with compost greatly increased soil pH values and facilitated switchgrass shoot masses up to almost 2.5 g (Fig. 4). Mixing in poultry litter biochar produced mixed impacts on soil pH and switchgrass shoot mass results. We found that incorporating 5% poultry litter biochar raised soil pH values to > 6, which corresponded to significant reductions in shoot mean masses (Table 4). Ideally, switchgrass growth in soil was optimal after treatment with 2.5% poultry litter biochar and either 2.5% or 5% compost treatment. After treatment with 5% poultry litter biochar, it was possible that switchgrass growth may have been limited by lower concentrations of plant-available P or other micronutrients due to precipitation at the higher soil pH values (Fig. 4) and/or by binding of P to Al and Ca in the biochar/compost (Table S2; Bohn et al. 1979). Viewing the clustering of data points between pH 5.3 and 5.9 that represents individual switchgrass above-ground biomass results vs. soil pH values indicates a suitable pH range for optimal switchgrass growth in this mine soil (Fig. 4). This finding is within the pH range of 5–8 pH for switchgrass establishment as reported by Hanson and Johnson (2005).

**Fig. 5:**
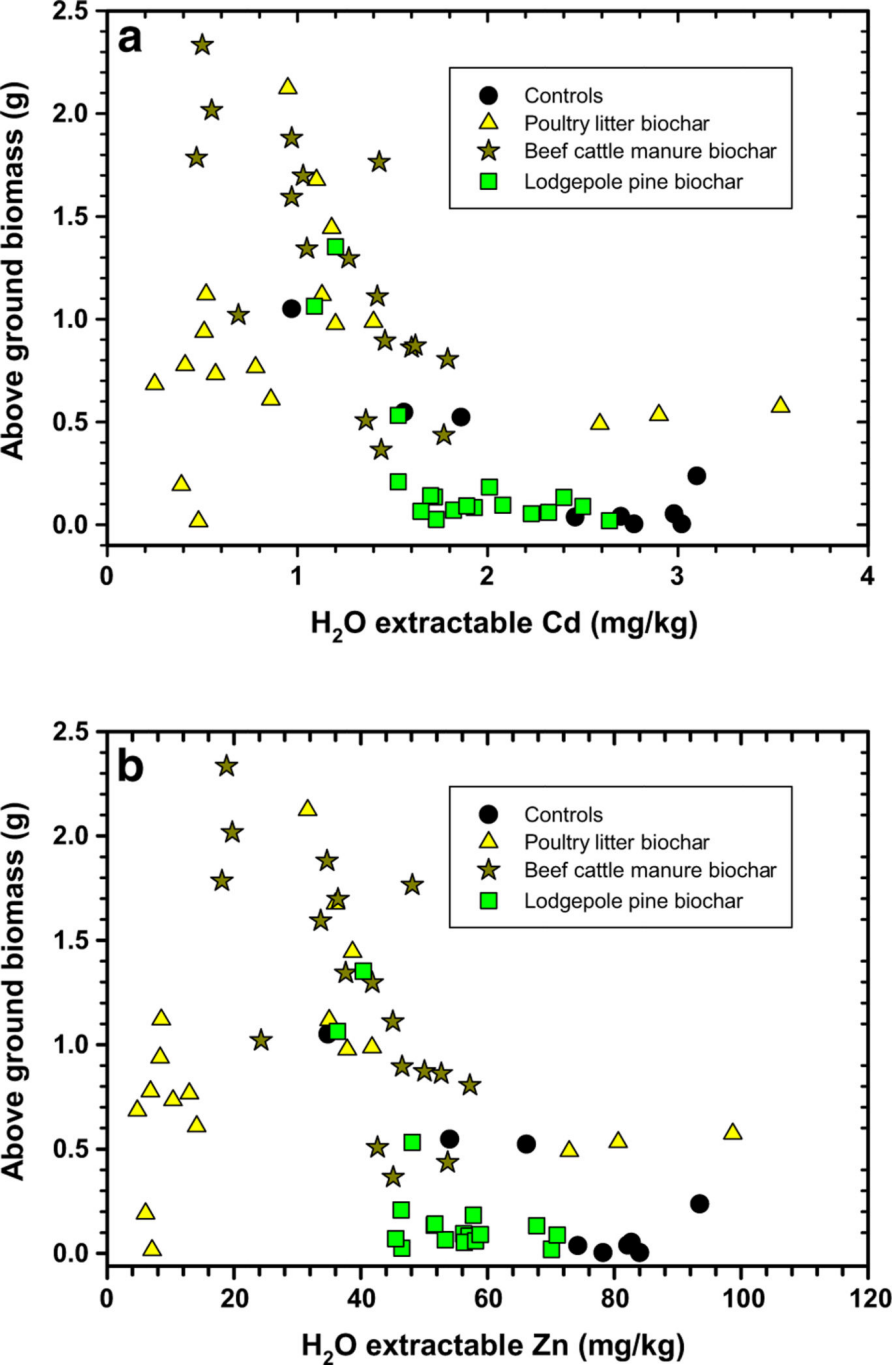
Water-extractable cadmium (Cd, **a**) and zinc (Zn, **b**) from compost + biochar-treated Tri-State Mine soil versus switchgrass above-ground biomass measured at end of study

**Fig. 6:**
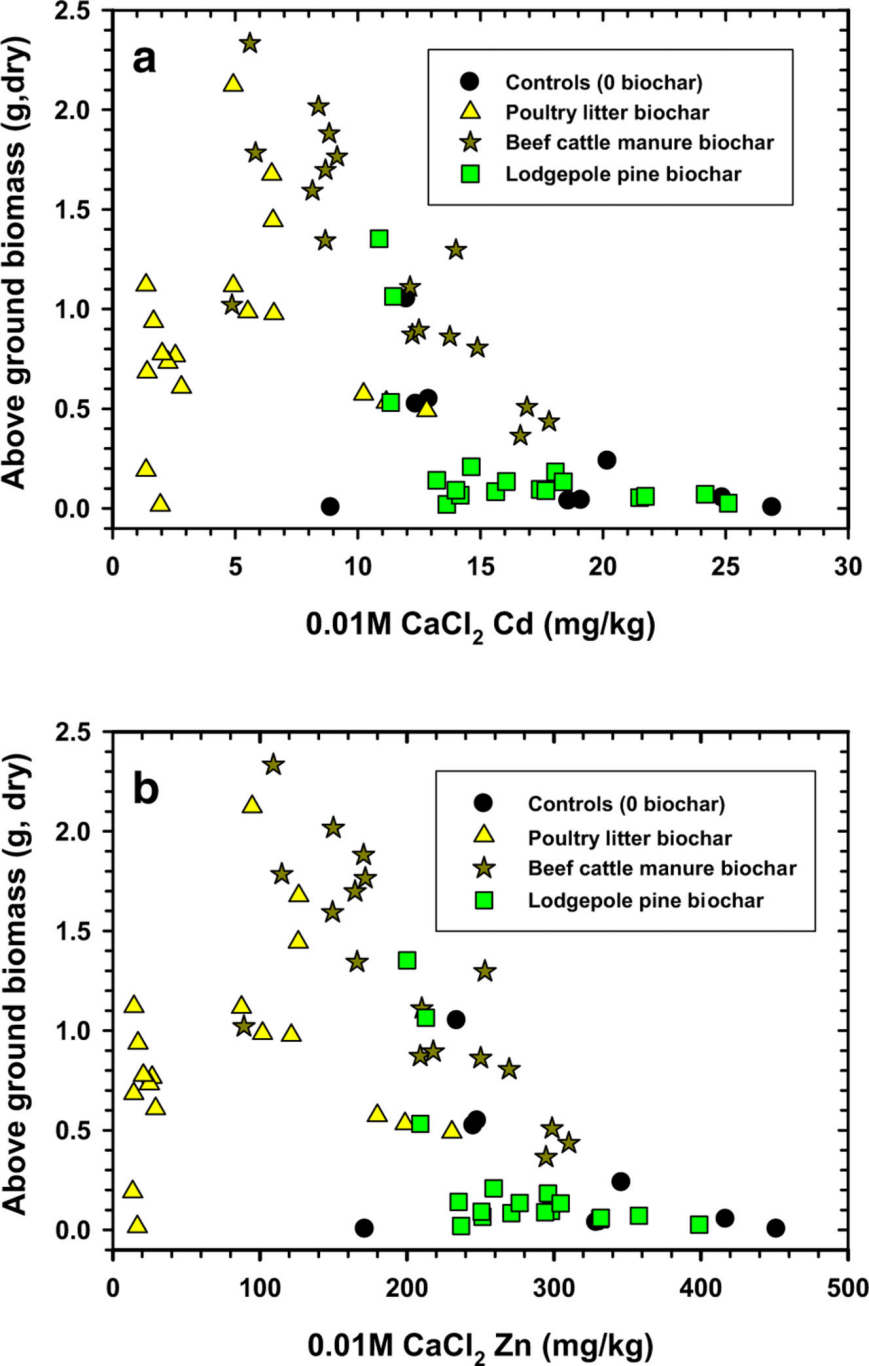
0.01 M CaCl_2_ extractable cadmium (Cd, **a**) and zinc (Zn, **b**) from compost + biochar-treated Tri-State Mine soil versus switchgrass above-ground biomass measured at end of study

Verification of improved switchgrass above-ground biomass production was linked to suppression of soil H_2_O extractable and bioavailable Cd and Zn concentrations (Figs. 5, 6). For switchgrass above-ground biomass to be > 1 g in this experiment, the biochars and compost needed to reduce H_2_O extractable Cd and Zn concentrations to < 1.5 and 50 mg/kg, respectively (Fig. 5). Maximum switchgrass growth was possible (or > 1.5 g) when the H_2_O extractable Cd and Zn concentrations were further reduced to < 1.0 and 40 mg/kg, respectively. The above-ground switchgrass biomass versus the 0.01 M CaCl_2_ extractable Cd and Zn concentration results followed a similar trend. Here, Cd and Zn concentrations should be reduced to < 10 and 150 mg/kg, respectively, for maximum switchgrass biomass production (> 1.5 g). Treatment of this soil with poultry litter biochar and beef cattle manure biochar and compost at certain applications will promote better switchgrass growth, meanwhile treatment with lodgepole pine biochar and compost produced poorer results. The lesser switchgrass above-ground biomass results are a consequence of the lodgepole pine biochar lacking the capability to reduce water and bioavailable Cd and Zn concentrations (Figs. 5, 6).

### 3.4 Cd and Zn concentrations measured in switchgrass shoots and roots

Cadmium is not an essential plant nutrient but is taken up by plants nonetheless (Kirkham 2006; Hasan et al. 2009). Plants, in general, can tolerate certain amounts of Cd, but Cd interferes with many physiological functions and can also induce phytotoxicity (Hasan et al. 2009; Khan et al. 2017). Reed et al (2002) reported that switchgrass (*Alamo* variety) grown in soil spiked with 200 mg/kg Cd decreased biomass accumulation by 95% with Cd concentrations measured at 900 mg/kg in root tissue while up to 100 mg/kg Cd was measured in switchgrass leaf tissue. In their study, the Cd concentrations in switchgrass roots and stems varied considerably with soil pH with greater plant concentrations measured when the experiments were conducted at low soil pH (4.01). Opposite to Cd, Zn is a plant micronutrient that is important for cellular enzymatic functions, protein production, and membrane integrity (Marschner 1995). When grown in soils with high Zn contents, plants can store more Zn in their roots than in the above-ground biomass, i.e., it is not readily translocated (Godbold et al. 1984). If grown in soils with excessive Zn levels, however, plants growth is inhibited, due to soil Zn toxicity causing root apical meristem necrosis, and eventually plant death follows. Zn toxicity levels in plants vary greatly, but Marschner (1995) reported that critical toxicity levels in plant leaves occur between 100 to more than 300 mg/kg.

**Fig. 7:**
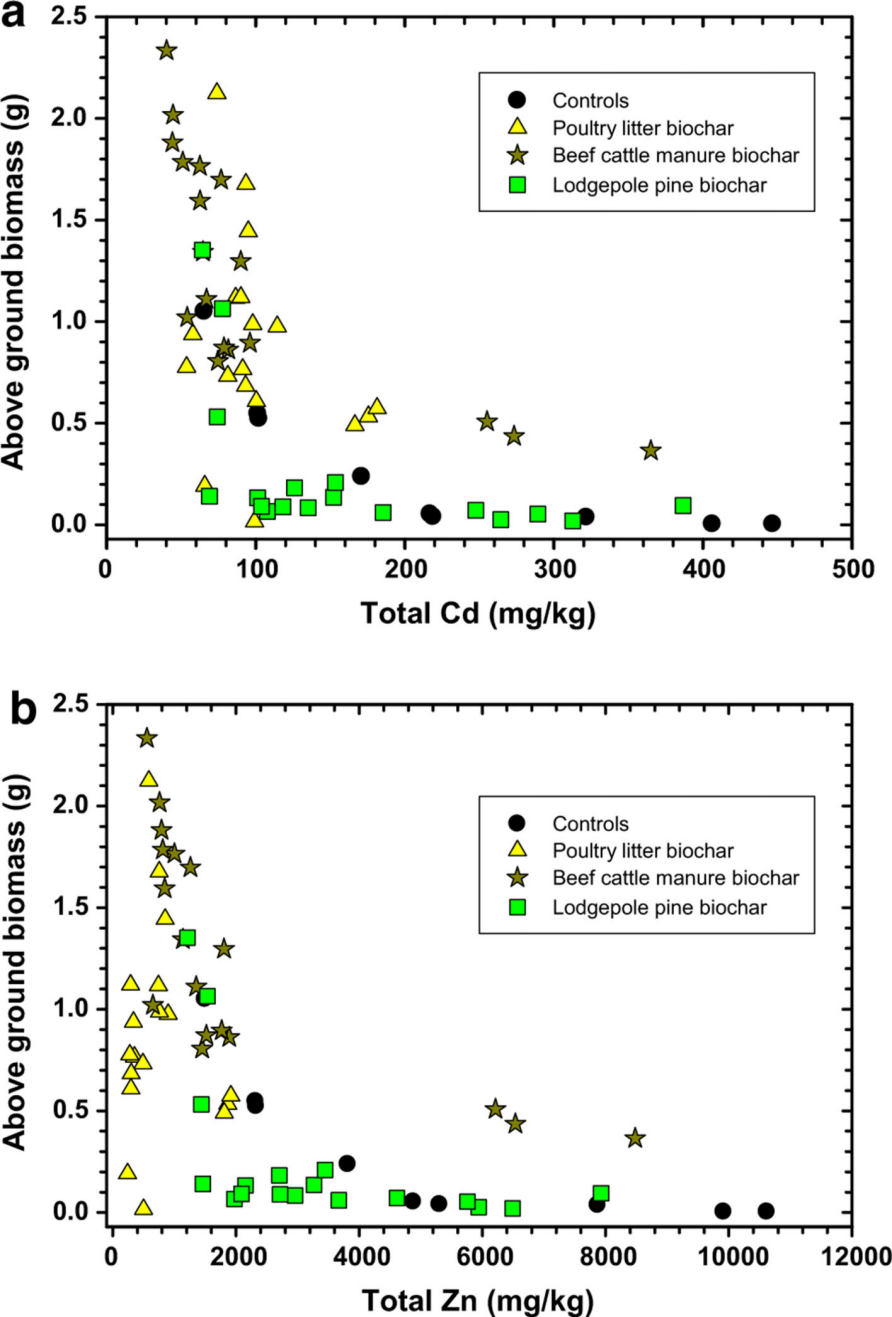
Total cadmium (Cd, **a**) and zinc (Zn, **b**) metal concentrations measured in switchgrass above-ground biomass at end of study

Figure 7 shows the overall relationship between total Cd (a) and total Zn (b) measured in switchgrass above-ground biomass as a function of biochar/compost treatment. The lowest total Cd measured (< 100 mg/kg) in switchgrass above-ground biomass occurred in plants grown in soil treated with biochar produced from poultry litter and beef cattle manure plus compost. Results presented in Table 5 support the significant decline in mean total Cd concentrations in switchgrass shoots after treatment with these two biochars. In fact, total Cd concentrations measured in switchgrass shoots were reduced by 74% and 64%, respectively, after treatment with 5% biochar produced from poultry litter and beef cattle manure feedstocks and 5% compost (Table 5). This agrees with previous results that showed reduced H_2_O and bioavailable-Cd concentrations as in Figs. 1 and 2. This trend is linked to an increase in soil pH following poultry litter and beef cattle manure biochar plus compost that reduces Cd solubility (Reed et al. 2002; Kirkham 2006). Biochar produced from lodgepole pine ± compost presented an inferior choice to suppress Cd assimilation because between 60 and 385 mg/kg were measured in switchgrass above-ground biomass (Fig. 7a). As shown in Table 5, there were some minor decreases in mean total Cd measured in switchgrass shoots, but the reduction was only about 35% when treated with 5% lodgepole biochar plus 5% compost as compared to the control at 0% biochar/0% compost (Table 5).

Total Zn concentrations in switchgrass above-ground biomass (Fig. 7b) followed a similar trend as observed with total Cd—biochars that caused greater increases in soil pH were more successful at reducing total Zn uptake. Mean total Zn concentrations were reduced between 81% and 94%, respectively, after soils were treated with biochar produced from beef cattle manure and poultry litter feedstocks plus 5% compost as compared to the control without any amendments (Table 5). Less total Zn was measured in switchgrass above-ground biomass after soils were treated with biochars produced from poultry litter and beef cattle manure feedstocks compared to lodgepole pine biochar (± compost). Treatment of soil with 2.5% lodgepole pine biochar plus 5% compost produced significant reductions in total Zn in shoots of 30% as compared to the mean treated with only 5% compost. Compost added alone to the soil was itself an effective amendment to reduce total Cd and Zn in shoots. Application of 5% compost alone was able to significantly reduce total Cd and Zn concentrations by about 75% in shoots compared to the untreated control (Table 5). The benefit of switchgrass containing lower total Cd and Zn concentrations is highlighted in Fig. 7a, b, which shows greater switchgrass above-ground biomass production after reduced uptake of these two metals. Antidotally, if the Tri-State Mining District site were to be reclaimed using the above-mentioned techniques, plants grown on site may contain lower Cd and Zn concentrations and thus be less of an issue to grazing wildlife in terms of food chain conclusions.

**Fig. 8:**
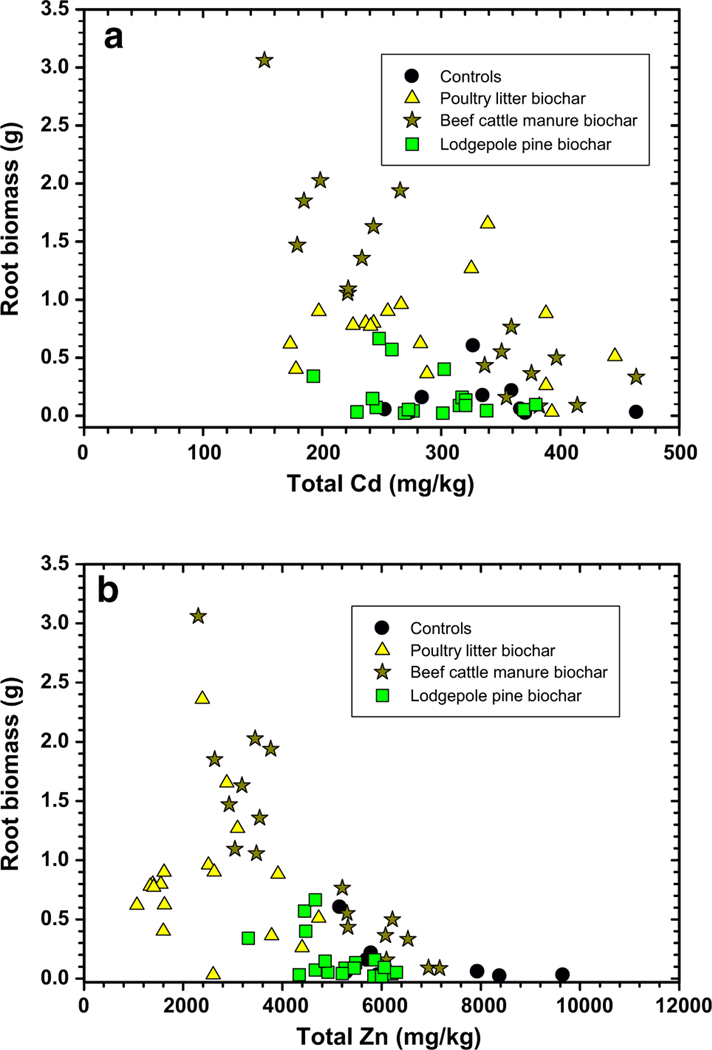
Total cadmium (Cd, **a**) and zinc (Zn, **b**) metal concentrations measured in switchgrass roots at end of study

Total Cd and Zn measured in switchgrass roots is shown in Fig. 8a, b. Total Cd in roots as a function of biochar treatment shows a complex pattern with some minor differentiation between treatments. Soil treated with biochar produced from beef cattle manure and poultry litter feedstocks cause significant reductions in total Cd and Zn contents in roots, but the significance of the reduction was dependent on quantities of biochar and compost applied (Table 6). In soils treated with 5% beef cattle manure biochar and poultry litter biochar and 5% compost, the largest reductions in mean total Cd and Zn concentrations measured in roots occurred. Soils treated with lodgepole pine biochar and compost showed significant reductions at application of 2.5% on total Zn in roots, but the treatments had no significant impact on means of total Cd in switchgrass roots (Table 6). Obviously, treatment of this mine soil with lodgepole pine biochar and compost would not be the most efficient biochar available for reduced metal uptake by switchgrass.

## 4 Conclusions

Biochar and compost amendments have emerged as potential remediation agents for sequestering heavy metals in mine spoils or in mine-impacted soils. This study evaluated the three biochar types produced from poultry litter, beef cattle manure, and lodgepole pine feedstocks, mixed into metal-contaminated soils with or without compost, on their ability to sequester Cd and Zn to levels that would allow switchgrass growth in a mine-impacted soil. Key findings were that the biochars differed greatly in their ability to reduce H_2_O extractable and bioavailable Cd and Zn—poultry litter and beef cattle manure biochar were more effective at reducing extractable Cd and Zn concentrations than lodgepole pine biochar. This condition was related to the ability of these two biochars to raise soil pH to > 5 which reduced the solubility of Cd and Zn and hence lowered their extractable concentrations. The greatest switchgrass and above-ground biomass and root production occurred in soils treated with 2.5% and 5% biochar from poultry litter and beef cattle manure plus 5% compost. In general, compost by itself was able to reduce soil-extractable Cd and Zn concentrations and total Cd and Zn in switchgrass shoots particularly at the 5% application rate. This study corroborates the finding that biochars should be carefully designed for their ability to modify soil conditions (i.e., increase pH, etc.) to greatly reduce bioavailable Cd and Zn concentrations in mine-impacted soils. If adopted, this paradigm can result in an optimized selection and application of the most efficient biochar in a mine spoil remediation plan.

## Supplementary Material

S1

## Figures and Tables

**Table 1:** Mean H_2_O-extractable Cd and Zn concentrations from Tri-State Mine soil after compost and biochar addition (*n* = 3 per treatment)

Compost (%)	Beef cattle manure biochar				Poultry litter biochar				Lodgepole pine biochar			
			
	0	2.5	5	*X*	0	2.5	5	*X*	0	2.5	5	*X*

H_2_O Cd (mg/kg)												
0	2.9 a, A	1.5 b, A	1.5 b, A	1.98 A	2.9 a, A	3.0 a, A	0.7 b, A	2.2 A	2.9 a, A	2.2 b, A	2.1 b, A	2.4 A
2.5	2.8 a, A	1.6 b, A	1.0 c, AB	1.8 A	2.8 a, A	1.2 b, B	0.5 c, A	1.5 B	2.8 a, A	1.7 b, AB	2.3 ab, A	2.3 A
5	1.5 a, B	1.0 ab, A	0.6 ab, A	1.0 B	1.5 a, B	1.2 a, B	0.4 b, A	1.0 C	1.5 a, B	1.3 a, B	1.8 a, a	1.5 B
*X*	2.5 a	1.4 b	1.0 c		2.4 a	1.8 b	0.5 c		2.4 a	1.7 b	2.1 c	
Source of Variation		*P*			*P*				*P*			
Biochar (*x*)		< 0.001			< 0.001				< 0.021			
Compost (*y*)		< 0.001			< 0.001				< 0.001			
*x* × *y*		0.027			< 0.001				0.031			

Compost (%)	Beef cattle manure biochar				Poultry litter biochar				Lodgepole pine biochar			
			
	0	2.5	5	*X*	0	2.5	5	*X*	0	2.5	5	*X*

B. H_2_O Zn (mg/kg)												
0	81.6 a, A	47.1 b, A	47.2 b, A	58.6 A	81.6 a, A	84.0 a, A	12.5 b, A	59.4 A	81.6 a, A	57.6 b, A	53.3 b, A	64.2 A
2.5	83.3 a, A	50.6 b, A	33.9 c, AB	55.9 A	83.3 a, A	37.5 b, B	7.4 c, A	42.7 B	83.3 a, A	51.7 b, A	65.5 b, A	66.8 A
5	51.7 a	36.2 b, A	20.4 b, B	361. B	51.7 a, B	36.1 b, B	6.4 c, A	31.4 C	51.7 a, B	41.7 a, A	54.6 a, A	49.3 B
*X*	72.2 a	44.7 b	33.8 c		72.1 a	52.6 b	8.7 c		72.1 a	50.3 b	57.8 a	
Source of Variation		P			P				P			
Biochar (*x*)		< 0.001			< 0.001				< 0.001			
Compost (*y*)		< 0.001			< 0.001				< 0.001			
*x* × *y*		0.142			< 0.001				0.037			

Lower case letter indicates significant differences among mean values between columns, while capital letter indicates significant differences among mean values within a column using a two-way ANOVA at a *P* < 0.05 level of significance

**Table 2:** Mean pH of Tri-State Mine soil after compost and biochar addition (*n* = 3 per treatment)

Compost (%)	Beef cattle manure biochar				Poultry litter biochar				Lodgepole pine biochar			
			
	0	2.5	5	*X*	0	2.5	5	*X*	0	2.5	5	*X*

0	4.40 a, A	5.07 b, A	5.31 c, A	4.93 A	4.40 a, A	5.46 b, A	6.33 c, A	5.39 A	4.40 a, A	4.37 a, A	4.77 a, A	4.42 A
2.5	4.69 a, B	5.19 b, A	5.61 c, B	5.16 B	4.69 a, B	5.58 b, A	6.53 c, A	5.60 B	4.69 a, B	4.77 a, B	4.89 b, B	4.78 B
5	5.10 a, C	5.28 a, A	5.92 b, C	5.43 C	5.10 a, C	5.85 b, B	6.61 c, B	5.85 C	5.10 a, C	5.10 a, C	5.05 a, c	5.09 C
*X*	4.73 a	5.20 b	5.61 c		4.73 a	5.63 b	6.49 c		4.73 ac	4.75 bc	4.81 b	
Source of Variation		*P*			*P*				*P*			
Biochar (*x*)		< 0.001			< 0.001				< 0.021			
Compost (*y*)		< 0.001			< 0.001				< 0.001			
*x* × *y*		0.025			0.018				0.009			

Lower case letter indicates significant differences among mean values between columns, while capital letter indicates significant differences among mean values within a column using a two-way ANOVA at a *P* < 0.05 level of significance

**Table 3:** Mean 0.01 M CaCl_2_-extractable Cd and Zn concentrations from Tri-State Mine soil after compost and biochar addition (*n* = 3 per treatment)

Compost (%)	Beef cattle manure biochar				Poultry litter biochar				Lodgepole pine biochar			
			
	0	2.5	5	*X*	0	2.5	5	*X*	0	2.5	5	*X*

A. 0.01 M CaCl_2_ Cd (mg/kg)												
0	20.2 a, A	17.1 ab, A	12.3 b, A	16.5 A	20.2 a, A	11.4 b, A	2.5 c, A	11.4 A	20.2 a, A	18.7 a, A	22.5 a, A	20.5 A
2.5	19.3 a, A	14.2 ab, AB	8.6 b, A	14.0 A	19.3 a, A	6.5 b, A	1.9 b, A	9.2 AB	19.3 a, A	15.4 a, A	18.0 a, AB	17.6 A
5	12.4 a, B	8.7 a, B	5.4 a, A	8.9 B	12.4 a, B	5.1 b, A	1.4 b, A	6.3 B	12.4 a, A	11.2 a, A	13.8 a, B	12.5 B
*X*	17.3 a	13.3 b	8.8 c		17.3 a	7.7 b	1.9 c		17.3 a	15.1 a	18.1 a	
Source of Variation		*P*			*P*				*P*			
Biochar (*x*)		< 0.001			< 0.001				0.267			
Compost (y)		< 0.001			0.016				0.001			
*x* × *y*		0.886			0.297				0.937			

Compost (%)	Beef cattle manure biochar				Poultry litter biochar				Lodgepole pine biochar			
			
	0	2.5	5	*X*	0	2.5	5	*X*	0	2.5	5	*X*

B. 0.01M CaCl_2_ Zn (mg/kg)												
0	346 a, A	301 ab, A	212 b, A	287 A	346 a, A	203 b, A	27 c, A	192 A	346 a, A	311 a, A	340 a, A	332 A
2.5	335 a, A	257 ab, AB	157 b, A	250 A	335 a, A	124 b, A	18 c, A	159 AB	335 a, A	269 a, A	298 a, A	300 A
5	242 a, A	167 ab, B	104 b, A	171 B	242 a, A	94 b, A	14 b, A	117 B	242 a, A	207 a, A	245 a, A	232 B
*X*	308 a	242 b	158 c		308 a	141 b	19 c		308 a	262 a	294 a	
Source of Variation		*P*			*P*				*P*			
Biochar (*x*)		< 0.001			< 0.001				0.259			
Compost (*y*)		< 0.001			< 0.022				0.006			
*x* × *y*		0.911			0.337				0.975			

Lower case letter indicates significant differences among mean values between columns, while capital letter indicates significant differences among mean values within a column using a two-way ANOVA at a *P* < 0.05 level of significance

**Table 4:** Switchgrass mean shoot and root growth from Tri-State Mine soil after compost and biochar additions (*n* = 3 per treatment)

Compost (%)	Beef cattle manure biochar				Poultry litter biochar				Lodgepole pine biochar			
			
	0	2.5	5	*X*	0	2.5	5	*X*	0	2.5	5	*X*

A. shoots (g, dry)												
0	0.020 a, A	0.436 a, A	0.959 b, A	0.472 A	0.020 a, A	0.532 a, A	0.703 a, A	0.418 A	0.203 a, A	0.470 a, A	0.062 a, A	0.043 A
2.5	0.105 a, A	0.988 b, B	1.792 c, B	0.962 B	0.105 a, A	1.366 b, B	0.577 a, A	0.682 AB	0.105 a, A	0.140 a, A	0.135 a, A	0.127 A
5	0.707 a, B	1.641 b, C	1.713 b, B	1.354 C	0.707 a A	1.641 b, C	1.713 b, B	1.354 B	0.707 a, B	0.983 a, B	0.099 b, A	0.596 B
*X*	0.277 a	1.022 b	1.488 c		0.277 a	1.10 b	0.648 c		0.277 a,c	0.391 a	0.0987 b,c	
Source of Variation		*P*			*P*				*P*			
Biochar (*x*)		< 0.001			< 0.001				0.009			
Compost (*y*)		< 0.001			0.021				< 0.001			
*x* × *y*		0.095			0.086				0.002			

Compost (%)	Beef cattle manure biochar				Poultry litter biochar				Lodgepole pine biochar			
			
	0	2.5	5	*X*	0	2.5	5	*X*	0	2.5	5	*X*

B. roots (g, dry)												
0	0.034 a, A	0.112 a, A	0.440 a, A	0.195 A	0.034 a, A	0.38 ab, A	0.832 b, A	0.415 A	0.034 a, A	0.0283 a, A	0.053 a, A	0.0384 A
2.5	0.086 a, A	0.543 a, A	1.486 c, B	0.705 B	0.086 a, A	1.268 b, B	0.477 a, A	0.611 A	0.086 a, A	0.10 a, A	0.107 a, A	0.0978 A
5	0.324 a, A	1.674 b, B	2.002 b, B	1.333 C	0.324 a, A	1.407 b, B	0.599 a, A	0.777 A	0.324 a, B	0.546 b, B	0.177 a, A	0.349 B
*X*	0.148 a	0.776 b	1.309 c		0.148 a	1.018 b	0.636 c		0.148 a	0.225 a	0.112 a	
Source of Variation		*P*			*P*				*P*			
Biochar (*x*)		< 0.001			< 0.001				0.116			
Compost (*y*)		< 0.001			0.119				< 0.001			
*x* × *y*		0.038			0.034				0.044			

Lower case letter indicates significant differences among mean values between columns, while capital letter indicates significant differences among mean values within a column using a two-way ANOVA at a *P* < 0.05 level of significance

**Table 5:** Total Cd and Zn in switchgrass shoots grown in Tri-State Mine soil after compost and biochar additions (*n* = 3 per treatment)

Compost (%)	Beef cattle manure biochar				Poultry litter biochar				Lodgepole pine biochar			
			
	0	2.5	5	*X*	0	2.5	5	*X*	0	2.5	5	*X*

A. total Cd in shoots (mg/kg)												
0	356 a, A	297 a, A	80 b, A	245 A	356 a, A	174 b, A	91 b, A	207 A	356 a, A	321 a, A	240 a, A	306 A
2.5	237 a, B	85 b, B	56 b, A	126 B	237 a, B	101 b, A	70 a, A	136 A	237 a, B	147 ab, B	115 b, B	166 B
5	89 a, C	61 a, B	48 a, A	66 C	89 a, C	86 a, A	83 a, A	86 A	89 a, C	72 a, B	93 a, B	85 C
*X*	228 a	148 b	61 c		228 a	120 b	81 b		228 a	180 a, b	149 b	
Source of Variation		*P*			*P*				*P*			
Biochar (*x*)		< 0.001			< 0.001				0.028			
Compost (*y*)		< 0.001			< 0.001				< 0.001			
*x* × *y*		0.005			0.005				0.267			

Compost (%)	Beef cattle manure biochar				Poultry litter biochar				Lodgepole pine biochar			
			
	0	2.5	5	*X*	0	2.5	5	*X*	0	2.5	5	*X*

B. total Zn in shoots (mg/kg)												
0	8469 a, A	7016 a, A	1546 b, A	5677 A	8469 a, A	1864 b, A	411 b, A	3581 A	8469 a, A	6785 ab, A	4679 b, A	6644 A
2.5	5663 a, B	1715 a, B	868 b, A	2749 B	5663 a, B	833 b, A	369 b, A	2288 A	5663 a, B	3225 ab, B	2523 b, A	3804 B
5	2046 a, C	1063 a, B	673 a, A	1260 C	2046 a, C	695 a, A	275 a, A	1005 B	2046 a, C	1391 a, B	1842 a, A	1760 C
*X*	5393 a	3265 b	1029 c		5393 a	1131 b	352 b		5393 a	3801 b	3015 b	
Source of Variation		*P*			*P*				*P*			
Biochar (*x*)		< 0.001			< 0.001				0.005			
Compost (*y*)		< 0.001			0.002				< 0.001			
*x* × *y*		0.006			0.005				0.207			

Lower case letter indicates significant differences among mean values between columns, while capital letter indicates significant differences among mean values within a column using a two-way ANOVA at a *P* < 0.05 level of significance

**Table 6:** Total Cd and Zn in switchgrass roots grown in Tri-State Mine soil after compost and biochar additions (*n* = 3 per treatment)

Compost (%)	Beef cattle manure biochar				Poultry litter biochar				Lodgepole pine biochar			
			
	0	2.5	5	*X*	0	2.5	5	*X*	0	2.5	5	*X*

A. total Cd in roots (mg/kg)												
0	299 a, A	383 a, A	383 a, A	355 A	299 a, A	374 b, A	225 a, A	299 A	299 a, A	282 a, A	249 a, A	276 A
2.5	388 a, A	377 a, A	218 b, B	328 A	388 a, A	350 a, A	264 a, A	334 A	388 a, A	334 a, A	340 a, A	354 B
5	323 a, A	228 b, B	186 b, B	246 B	323 a, A	265 a, A	233 a, A	274 A	323 a, A	269 a, A	257 a, A	283 A
*X*	337 a	329 a	262 b		377 a	330 a	241 b		337 a	295 a	282 a	
Source of Variation		*P*			*P*				*P*			
Biochar (*x*)		0.006			0.006				0.052			
Compost (*y*)		< 0.001			0.143				0.004			
*x* × *y*		< 0.001			0.341				0.923			

Compost (%)	Beef cattle manure biochar				Poultry litter biochar				Lodgepole pine biochar			
			
	0	2.5	5	*X*	0	2.5	5	*X*	0	2.5	5	*X*

B. total Zn in roots (mg/kg)												
0	6534 a, AB	6737 a, A	5717 a, A	6329 A	6534 a, AB	4303 b, A	1516 c, A	4188 A	6534 a, AB	5805 a, A	4640 a, A	5729 AB
2.5	7797 a, B	5833 b, A	3216 c, B	5616 A	7797 a, B	3295 b, A	1668 c, A	4254 A	7797 a, A	4526 b, A	5660 b, A	6421 A
5	5552 a, A	3563 b, B	2659 b, B	3925 B	5552 a, A	2506 b, A	1542 b, A	3200 A	5552 a, B	4640 a, A	4459 a, A	4846 B
*X*	6628 a	5378 a	3864 b		6628 a	3368 b	1575 c		6628 a	5448 b	4920 b	
Source of Variation		*P*			*P*				*P*			
Biochar (*x*)		< 0.001			< 0.001				< 0.004			
Compost (*y*)		< 0.001			0.054				0.009			
*x* × y		0.029			0.151				0.551			

Lower case letter indicates significant differences among mean values between columns, while capital letter indicates significant differences among mean values within a column using a two-way ANOVA at a *P* < 0.05 level of significance
