# Supplementary material for: Biochar compost blends facilitate switchgrass growth in mine soils by reducing Cd and Zn bioavailability
Source: Biochar. Author manuscript; Available in PMC 2022 Mar 22. (PMC8939468; doi:10.1007/s42773-019-00004-7)
Supplement: S1 [file NIHMS1533147-supplement-S1.docx]

**Supplementary Tables & Figures:**

Fig. S1. Collection site of Tri-State Mine soil (A), back hoe collection method (B), and redoxy-morphic features of soil (C).

Table S1. Chemical properties of Tri-State mine soil.

Table S2. Chemical and physical properties of compost and biochars.

Table S3. Treatments used in Switchgrass greenhouse experiment.


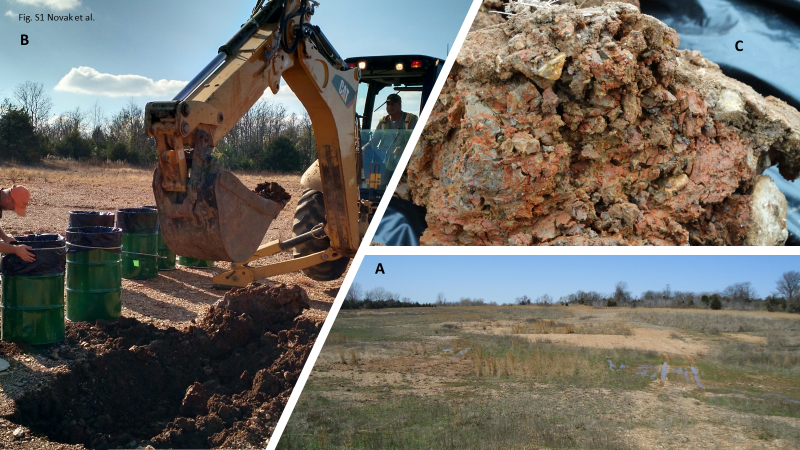


Fig. S1 Novak et al.

| Table S1. Chemical properties of Tri-State Mine soil (means of n = 3; standard deviation in parentheses; ND = not determined; 0 value = below detection) | | | |
| --- | --- | --- | --- |
|  |  | Extractable metal (mg/kg) | |
| Element | Total metal (mg/kg)† | H_2_O | 0.01 M CaCl_2_ |
| Al | ND | 4.40 (3.87) | 11.36 (1.56) |
| Cl | ND | ND | ND |
| Cd | 72.2 (2.7) | 5.73 (0.98) | 50.45 (0.40) |
| Cr | ND | 0 | 0.12 (0.01) |
| Cu | 66.5 (2.5) | 0.22 (0.07) | 2.17 (0.02) |
| Fe | ND | 10.57 (2.11) | 12.65 (1.45) |
| K | 711 (25) | 26.18 (3.47) | 59.48 (3.35) |
| Mg | 355 (45) | 4.53 (1.38) | 36.49 (0.97) |
| Mn | 72 (5.7) | 2.48 (0.72) | 21 (0.9) |
| Na | ND | 22.25 (4.01) | 25.58 (4.24) |
| Ni | 7.6 (0.3) | 0.18 (0.01) | 0.45 (0.01) |
| P | 168 (4) | 3.89 (0.06) | 1.43 (1.30) |
| Pb | 23.5 (0.7) | 0 | 0 |
| SO_4_ | ND | 152.6 (19.4) | 112.8 (17.4) |
| Zn | 2225 (12) | 141.0 (25.7) | 782 (13) |

†samples digested using 4M HNO_3_

| Table S2. Chemical and physical properties of compost and biochars (dry-basis) | | | | |
| --- | --- | --- | --- | --- |
| 1. Ultimate and proximate analysis | | | | |
|  | Beef cattle manure | | Lodgepole pine | Poultry litter |
| Measurement (%) | Compost | biochar | biochar | biochar |
| C | 17.5 | 13.8 | 90.5 | 37.4 |
| H | 1.9 | 0.7 | 2.4 | 2.8 |
| O | 10.5 | 1.4 | 3.2 | 13.0 |
| N | 1.6 | 1.0 | 0.7 | 4.2 |
| S | 0.09 | 0.02 | < 0.001 | 0.07 |
| Ash | 68.4 | 83.1 | 3.2 | 42.5 |
| Fixed C | 6.1 | 9.4 | 82.5 | 21.2 |
| Volatile matter | 25.5 | 7.5 | 14.3 | 36.3 |
|  |  |  |  |  |
| pH | 6.8 | 9.5 | 9.7 | 9.1 |
| O/C | 0.46 | 0.07 | 0.03 | 0.26 |
| H/C | 1.29 | 0.60 | 0.32 | 0.89 |
|  |  |  |  |  |
| 1. Elemental analysis of ash (%, ash wt basis) | | | | |
| Al | 3.0 | 2.9 | 0.9 | 0.9 |
| As | < 0.005 | < 0.005 | 0.1 | < 0.005 |
| Ca | 3.0 | 2.8 | 11.8 | 11.6 |
| Cd | < 0.005 | < 0.005 | < 0.005 | < 0.005 |
| Cl | < 0.01 | < 0.01 | 0.6 | 5.6 |
| Cr | < 0.005 | < 0.005 | 0.15 | 0.01 |
| Cu | 0.005 | 0.005 | 0.26 | 0.4 |
| Fe | 1.43 | 1.41 | 1.13 | 1.11 |
| K | 2.2 | 2.13 | 3.9 | 18.0 |
| Mg | 0.93 | 0.90 | 2.6 | 3.9 |
| Mn | 0.09 | 0.10 | 0.35 | 0.28 |
| Na | 0.31 | 0.30 | 1.1 | 4.5 |
| Ni | 0.005 | 0.006 | 0.03 | 0.016 |
| P | 0.67 | 0.68 | 0.4 | 8.6 |
| Pb | < 0.005 | < 0.005 | 0.09 | < 0.005 |
| S | 0.25 | 0.22 | 0.58 | 4.9 |
| Si | 77.6 | 77.2 | 18.2 | 8.4 |
| Zn | 0.03 | 0.03 | 0.09 | 0.23 |

| Table S3. Treatments used in Switchgrass greenhouse experiment. | |
| --- | --- |
| % application rate (w/w) | |
| Biochar | Compost |
| 0 | 0 |
| 0 | 2.5 |
| 0 | 5 |
| 2.5 | 0 |
| 2.5 | 2.5 |
| 2.5 | 5 |
| 5 | 0 |
| 5 | 2.5 |
| 5 | 5 |
